# WDR82 suppresses breast cancer progression by inhibiting ERK-driven chemokine expression and neutrophil infiltration

**DOI:** 10.7150/ijbs.135015

**Published:** 2026-05-29

**Authors:** Qinyi Yu, Jiang Yang, Fengjiao Lu, Wenxiu Liu, Zeyao Han, Yuanchao Bao, Xiaorui Xu, Jialei Xu, Wanfeng Gao, Boyi Cong, Yangyang Chai, Xuetao Cao

**Affiliations:** 1 Institute of Immunology, Zhejiang University School of Medicine, Hangzhou 310058, China.; 2 Department of Immunology, Institute of Basic Medical Sciences & School of Basic Medicine, Chinese Academy of Medical Sciences & Peking Union Medical College, Beijing 100005, China.; 3 State Key Laboratory of Medicinal Chemical Biology, Institute of Immunology, College of Life Sciences, Nankai University, Tianjin 300071, China.

**Keywords:** breast cancer, WDR82, antitumor effect, immunosuppressive TME, neutrophil, chemokines, MEK1/2, ERK

## Abstract

Elucidating tumor-intrinsic mechanisms orchestrating immunosuppressive tumor microenvironment (TME) and cancer immunoevasion is essential for overcoming cancer immunotherapy resistance and developing novel therapeutic strategies. WDR82, a member of the WD-40 protein family, exhibits context-dependent regulation of cancer with undefined role in breast cancer and immunosuppressive TME. Here we identify WDR82 as a critical tumor suppressor governing immune surveillance by restraining the ERK-chemokine-neutrophil axis. WDR82 expression is downregulated in human breast cancer and correlates with poor prognosis. Although WDR82 promotes tumor cell proliferation *in vitro*, it suppresses tumor growth *in vivo* exclusively in immunocompetent hosts by enhancing neutrophil infiltration and CD8⁺ T cell exhaustion, creating an immunosuppressive TME. Neutrophil depletion abolishes WDR82's tumor-suppressive effect, confirming the functional dependency on this axis. Mechanistically, WDR82 directly binds MEK1/2 to disassemble c-RAF-MEK binding and inactivate ERK signaling. *Wdr82* deletion restores ERK-dependent tumor cell CXCL2 and CXCL7 production to promote CXCR2-mediated neutrophil recruitment in the TME. Importantly, intratumoral WDR82-expressing adenovirus delivery reverses neutrophil accumulation and T cell exhaustion, suppressing tumor progression. Our findings uncover a previously unrecognized mechanism by which WDR82 suppresses MEK-ERK signaling, ERK-dependent chemokines production and neutrophil infiltration, consequently relieving immunosuppressive TME. This work establishes a promising therapeutic strategy for breast cancer.

## Introduction

Breast cancer remains the leading cause of cancer-related mortality among women worldwide, with its incidence projected to rise substantially over the coming decades^1^. Although significant advances have been made in targeted therapies and immunotherapies^2^-^5^, the challenges persist, particularly in triple-negative breast cancer (TNBC) which lacks effective therapeutic targets and is characterized by a highly immunosuppressive TME^6^, ^7^. Identifying novel molecules that simultaneously suppress tumor cell-intrinsic properties and remodel the TME represents a promising strategy for designing new therapeutic approaches and improving therapeutic outcomes^8^-^11^.

WDR82, a core component of the SET1/COMPASS complex, has been implicated in diverse biological processes including epigenetic regulation, transcription termination, and cellular reprogramming^12^-^16^. Emerging evidence points to its context-dependent roles in cancer: while WDR82 promotes tumor proliferation in pediatric high-grade glioma via H3K4me3-mediated cyclin D1 activation^17^, it is significantly downregulated in colorectal and lung cancers and correlates with poor prognosis^18^-^20^. These observations suggest that WDR82 may function as either an oncogene or a tumor suppressor depending on the tumor type. However, its expression pattern, biological function, and potential role in modulating the immune landscape and affecting therapeutic outcome in breast cancer remain entirely unexplored.

Neutrophils, one of the most abundant immune cells in the TME, exert dual functions in tumor progression^21^. Accumulating evidence shows that tumor-associated neutrophils (TANs) predominantly acquire a pro-tumorigenic (N2) phenotype, promoting angiogenesis, immunosuppression, and resistance to both chemotherapy and immunotherapy^22^-^25^. Given the critical role of the TME in dictating breast cancer progression and treatment response^21^, ^26^, ^27^, understanding how tumor cell-intrinsic alterations influence neutrophil recruitment and polarization in TME is an important issue, which needs to be addressed.

By performing *in vivo* genome-wide CRISPR-Cas9 screening in mouse TNBC model, we previously uncovered numerous hits with tumor promoting potential, such as cancer cell CD28 promoting cancer immune escape, or tumor constraining potential, among which WDR82 stood out as a top candidate with tumor suppressor property^28^. In this study, we report that WDR82 suppresses breast cancer by governing neutrophil infiltration through disrupting ERK-CXCL signaling axis. By integrating clinical specimen analysis, murine orthotopic models, and multi-omics approaches, we uncover a previously unrecognized mechanism by which WDR82 restricts tumor progression: WDR82 directly binds to MEK1/2 to suppress ERK activation which subsequently inhibits CXCL2/CXCL7-mediated neutrophil chemotaxis. Importantly, we demonstrate that WDR82 expression is downregulated in human breast cancer and that its restoration via intratumoral adenoviral delivery effectively suppresses tumor growth *in vivo* by relieving the immunosuppressive TME. Our findings reveal WDR82 as a key regulator of tumor-immune crosstalk for breaking immune evasion and highlight its potential as a therapeutic target for breast cancer immunotherapy.

## Methods

### Cell lines

The cell lines 4T1 and EMT6 were obtained from American Type Culture Collection (ATCC) and cultured in RPMI-1640 Medium (Corning, 10-040-CV) supplemented with 10% FBS (Gibco,10099-1441C) and 1% penicillin-streptomycin (Gibco, 15140122). All cell lines were confirmed to be free of mycoplasma contamination.

### Mouse models

Six-to-eight-week-old female BALB/c or NOD/SCID mice were obtained from Beijing Vital River Laboratory Animal Technology Co., Ltd, and were bred in standard pathogen-free conditions with 12-h light-dark cycles.

All mice were randomly assigned to different experimental groups and animal experiments were performed as previously described^28^. For tumor inoculation, 5×10^5^ 4T1 cells in 100 μL PBS were orthotopically injected into the fourth mammary gland pad of mice. For adenovirus-mediated overexpression of WDR82, ad-WDR82 or ad-NC was injected intratumorally at 1×10^9^ PFU per day for three consecutive days after tumor volume reached 100 mm^3^. For neutrophil depletion, 200 μg anti-mouse Ly6G (BioXCell, BE0075) or Rat Isotype control IgG2a (BioXCell, BE0089) were injected intraperitoneally one day before tumor inoculation and 100 μg every three days later. The depletion efficacy was determined using Fluorescence-Activated Cell Sorting (FACS). Tumor volume was measured using the formula: Length×Width^2^/2. The mice were euthanized when the tumor volume reached 2000 mm^3^. All animal experiments were performed in accordance with protocols approved by the Institutional Animal Care and Use Committee of Nankai University (approval number: 2021-SYWDLL-000355 and 2026-SYDWLL-000724).

### Neutrophil isolation and culture

Mouse neutrophils were isolated from bone marrow using EasySep™ Mouse Neutrophil Cell Isolation Kit (STEMCELL Technologies, 19762). In brief, bone marrow was flushed from mouse femurs and tibias and centrifuged at 800g for 10 minutes. The cells were then resuspended using pre-cooled PBS and filtered through a 70 μm filter to obtain single cell suspension. Neutrophils were isolated using the isolation kit and cultured in RPMI-1640 supplemented with 10% FBS and 1% penicillin-streptomycin. The purity of isolated neutrophils was determined using FACS (CD45^+^CD11b^+^Ly6G^+^) to be over 90%. The isolated neutrophils were used immediately.

### Human samples

The tissue chips were gained from Shanghai OUTDO BIOTECH CO., LTD. All patients were diagnosed with breast cancer by pathologist and did not receive any treatment before surgery. Tumor stage was identified based on TNM staging system. All analyses of human samples were carried out in compliance with the relevant ethical regulations and approved by Shanghai OUTDO BIOTECH CO., LTD (approval number: YB M-05-02).

### Generation of *Wdr82* knockout cell lines using CRISPR-Cas9

For knockout of mouse *Wdr82* gene in 4T1 and EMT6 cells, two CRISPR single guide RNAs (sgRNAs) targeting the coding sequence were designed online (https://www.synthego.com/products/bioinformatics/crispr-design-tool). The sgRNAs were cloned into pSpCas9 (BB)-2A-GFP plasmids (PX458) (Addgene, plasmid 48138) using BbsI (Thermo Fisher Scientific, ER1012) respectively. Two plasmids containing the indicated sgRNAs were co-transfected into relevant cells using Lipofectamine 3000 Reagent (Invitrogen, L3000015) according to manufacturer's procedures. Meanwhile, the cells transfected with empty vector (px458) were used as control. GFP-positive cells were sorted into single cells by FACS (SONY, MA900) and seeded into 96-well plates for clonal expansion 24 hours post transfection. The efficiency of WDR82 knockout in cells were verified by Western blot analysis. The sgRNAs sequences are shown in Supplementary Table 1.

### Construction of WDR82-Flag expressing cell line

The cDNAs of mouse *Wdr82* (m*Wdr82*) were synthesized in GENWIZ and subcloned into the pcDNA3.1 control with a C-terminal Flag tag (pcDNA3.1-Flag, invitrogen) for recombinant expression. The plasmid was transfected into *Wdr82* deficient or control cells using Lipofectamine 3000 Reagent according to manufacturer's procedures. Meanwhile, the cells transfected with empty vector (pcDNA3.1-Flag) were used as control. After 48 hours of transfection, the transfected cells were screened using 2 mg/mL G418 (Beyotime, ST018) for 14 days. The efficiency of indicated gene expression was verified by Western blot analysis.

### Preparation and infection of mWDR82-expressing adenovirus

The cDNA of m*Wdr82* was subcloned into GV135 (Genechem) and then co-transfected into HEK293T cells with the packaging plasmid pBHG lox ΔE1, 3 Cre (Genechem) using Lipofectamine 2000 Reagent (Invitrogen, 11668-019) according to instructions. Cells were collected by low-speed centrifugation and subjected to three freeze/thaw cycles between -70°C to 37°C at 10-15 days post transfection. Subsequently, the viral supernatant was collected by centrifugation at 7000 g for 5 minutes at 4°C, purified using the Adeno-X™ Viral Purification Kit (Clontech, 631533) and stored at -80°C. The purified adenovirus was sterile and yielded a functional titer of 3×10^11^ PFU/mL, as determined by end-point dilution assay. Cells were infected with adenovirus for 48 hours, and then the expression efficiency was measured by Western blot analysis.

### Western blot

For Western blot analysis, whole-cell lysates were prepared in RIPA lysis buffer (Beyotime, P0013B) supplemented with EDTA-free protease inhibitor cocktail (MedChemExpress, HY-K0011), PMSF (Beyotime, ST506) and Phosphatase Inhibitor Cocktail A (Beyotime, P1082). The protein lysate was then boiled in 5×SDS-PAGE Sample Buffer (GenStar, E153-10) for 5 minutes and separated by SDS-PAGE. The samples were then transferred to a 0.45 μm nitrocellulose blotting membrane (Cytiva, 10600002) and blocked in 5% non-fat milk (BBI, A600669-0250) or Bovine Serum Albumin (Sigma-Aldrich, 900933-100G) in TBST for 1 hour at room temperature, followed by incubation with primary antibody in Universal Antibody Diluent (NCM, WB100B) at 4℃ overnight. After washing, membranes were incubated with secondary antibodies in Universal Antibody Diluent at room temperature for 1 hour and visualized using NcmECL Ultra (NCM, P10300). Primary antibodies used in this study include anti-WDR82 (Abcam, EPR27034-63), anti-Phospho-p44/42 MAPK (Erk1/2) (Thr202/Tyr204) (Cell signaling Technology, 4370T), anti-Phospho-MEK1/2 (Ser217/221) (Cell signaling Technology, 9154T), anti-Phospho-c-RAF (Ser338) (Cell signaling Technology, 9427T), anti-MEK1/2 (Cell signaling Technology, 9122S), anti-p44/42 MAPK (Erk1/2) (Cell signaling Technology, 4695T), anti-DYKDDDDK Tag (Cell signaling Technology, 14793T), anti-c-RAF (Cell signaling Technology, 53745T), anti-GAPDH (PTM Bio, PTM-6684) anti-β-Actin (PTM Biolabs, PTM-5018) and anti-α-Tubulin (PTM Biolabs, PTM-5442). Secondary antibodies used in this study include goat anti-Rabbit IgG-HRP (Absin, abs20040) and goat anti-Mouse IgG-HRP (Absin, abs20001). All primary antibodies were diluted at 1:1000 and secondary antibodies were diluted at 1:2000.

### RNA interference

Cells were co-transfected with siRNAs against the indicated gene or non-targeting control siRNAs using Lipofectamine™ RNAiMAX Transfection Reagent (Invitrogen, 13778150) according to manufacturer's procedure. RNA was extracted after 48 hours to detect knockdown efficiency through quantitative real-time PCR (qRT-PCR).

### RNA extraction and qRT-PCR

RNA was extracted from cells using RNAfast200 kit (Fastagen, 220011) following the standard protocol. cDNA was generated using RT Master Mix (TOYOBO, FSQ-201) and qRT-PCR was performed using SYBR® qPCR Master Mix (TOYOBO, QPK-201) on Real-time PCR instrument (Thermo Fisher Scientific, QuantStudio 7 Flex). *Actin* level was used to normalize relative gene expression levels. Primers used are shown in Supplementary Table 2.

### Co-immunoprecipitation (Co-IP)

Control, *Wdr82^-/-^* or *Wdr82^-/-^*+WDR82-Flag 4T1 cells treated with 20ng/mL EGF for 1 hour were lysed with IP lysis buffer (Beyotime, P0013) supplemented with Halt Protease and Phosphatase Inhibitor Cocktail (Thermo Fisher Scientific, 78442). Protein concentrations were measured using BCA assay (Thermo Fisher Scientific, 23225). Protein concentration was adjusted to 1 μg/μL and 200 μL soluble fraction was subjected to immunoprecipitation with 4 μL antibody recognizing c-RAF, Flag-tag or IgG at 4°C overnight. 30 μL Protein A/G Magnetic Beads (MedChemExpress, HY-K0202) prewashed with IP lysis buffer were added and incubated for another 3 hours at 4°C. Protein complex-containing beads were washed twice with medium salt washing buffer (0.5% NP-40, 2mM EDTA, 500mM NaCl, 20mM Tris-HCl (pH=7.0)) followed by twice with low salt washing buffer (0.5% NP-40, 2mM EDTA, 150mM NaCl, 20mM Tris-HCl (pH=7.0)). Proteins were boiled in 2× SDS loading buffers and analyzed using Western blot analysis.

### Cell viability assay

Indicated cells were seeded in 96-well plates at 3×10^3^ cells per well and cultured for 24, 48 and 72 hours. Cell viability was measured using CCK8 assay (Beyotime, C0041) according to the manufacturer's instructions. Briefly, supernatant was removed and 100μL fresh cell culture medium supplied with 10 μL WST-8 was added to each well and incubated at 37°C for 1 hour. The absorbance (optical density, OD) at 450 nm was measured.

### Colony-formation assay

Colony formation was performed as previously described^28^. Briefly, indicated cells were seeded into 6-well plates at 300 cells per well. After about 5-10 days of routine culturing, cells were stained using Crystal Violet Staining Solution (Beyotime, C0121). The number of colonies formed were counted.

### Transwell assay

For tumor cell transwell assay, cells were seeded into the upper chamber at 3×10^4^ cells per well. Serum-free RPMI-1640 medium was added to the upper chamber and RPMI-1640 medium supplemented with 10% FBS was added to the lower chamber. Cells were stained using Crystal Violet Staining Solution after 48 hours of incubation. For neutrophil transwell assay, cells were seeded into the upper chamber at 1×10^5^ cells per well. RPMI-1640 supplemented with 10% FBS and 1% penicillin-streptomycin was added to the upper chamber and supernatant collected from indicated cells was added into the lower chamber. Cells in the lower chamber were counted using Precision Count Beads (Biolegend, 424902) after 3 hours of incubation.

### Wound healing assay

Indicated cells were seeded into 6-well plates at 5×10^5^ cells per well and scratches were made with a 200 µL pipette tip when cells reached about 90% confluent. Cells were washed three times with PBS to discard the floating cells and cultured in serum-free RPMI-1640 medium. Images were taken using a light microscope with an attached digital camera at 0, 12 and 24 hours.

### Enzyme-linked immunosorbent assay (ELISA)

Supernatants from 5×10⁵ indicated cells cultured in 6-well plates were collected 24 hours after stimulation with 20 ng/mL EGF. Cytokine levels were assessed using ELISA kits for CXCL2 (JOT-EK2613Mo) and CXCL7 (JOT-EK2614Mo) following manufacturer's instructions.

### Flow cytometry

The primary tumors of tumor-bearing mice were harvested and processed as previously described^28^. Briefly, tumors were mechanically dissociated and digested with 1 mg/mL collagenase type I (Gibco, 17018029), 1 mg/mL collagenase type IV (Gibco, 17104019) and 0.3 mg/mL DNase I (Sigma-Aldrich, DN25) at 37℃ for 60 minutes and filtered through a 40 μm cell strainer to achieve a single-cell suspension. Erythrocytes were removed using erythrocyte lysate (Invitrogen, 00-4333-57). Cells were incubated with TruStain FcX (BioLegend, 101320) for 10 minutes at 4℃ to block non-specific staining and then incubated with the following antibodies for 30 minutes at 4℃ in the dark for further analysis or sorted using FACS (BD, LSRFortessa^TM^ X-20 or Sony, MA900). The antibodies included Zombie-Aqua (BioLegend, 423102), APC/Cyanine7 anti-CD45 (BioLegend, 157617), PerCP/Cyanine5.5 anti-CD3 (BioLegend, 100218), Brilliant Violet 605^TM^ anti-CD8 (BioLegend, 100744), Brilliant Violet 650^TM^ anti-CD4 (BioLegend, 100546), PE/Cyanine7 anti-CD49b (BioLegend, 103517), FITC anti-CD11b (BioLegend, 101205), Brilliant Violet 421^TM^ anti-Ly6G (BioLegend, 127627), PE anti-Ly6C (BioLegend, 128008), APC anti-F4/80 (BioLegend, 123116), PE/Cyanine7 anti-CD11c (BioLegend, 117318), PerCP/Cyanine5.5 anti-I-A/I-E (BioLegend, 107625), FITC anti-PD-1 (Biolegend, 135213), PE anti-LAG3 (Biolegend, 125207), APC anti-TIM3 (Biolegend, 134007) and Brilliant Violet 421^TM^ anti-CD19 (Biolegend, 152421), Brilliant Violet 421^TM^ anti-XCR1 (Biolegend, 148216).

### Multiplex immunofluorescence staining (mIF)

mIF was performed as previously described^28^. In brief, the tumor tissues were fixed in 4% Paraformaldehyde Fixative (Biosharp, BL539A), and paraffin-embedded sections (3-4 μm in thickness) were prepared according to routine operating procedures. mIF was performed using a Opal™ 7-Color Manual IHC Kit (Akoya Biosciences, NEL811001KT) following the manufacturer's instructions. Different primary antibodies were sequentially applied: WDR82 (1:1000, Abcam) and Ly6G (1:1000, Abcam) followed by HRP-conjugated secondary antibody incubation and tyramide signal amplification (TSA). The slides were microwave-treated after each cycle of TSA. Nuclei were stained with 4'-6'-diamidino-2-phenylindole (DAPI) for 10 min. The sample scanning, spectral unmixing and quantification of signals were conducted with the Vectra Polaris Automated Quantitative Pathology Imaging System (Akoya), using the Phenochart and InForm V.2.4 softwares (Akoya).

### RNA-sequencing

Indicated cells were collected and washed twice using cooled PBS buffer, RNA was extracted using TRIzol regent (Thermo Fisher Scientific, 15596018CN). mRNA was enriched from total RNA using Oligo(dT) beads. The extracted mRNA was then reverse-transcribed and barcoded. The barcoded cDNA was then amplified by PCR to generate sufficient mass for library construction. Sequencing libraries were constructed via end repair, A-tailing, adaptor ligation, and PCR, and sequencing was performed with Illumina HiSeq (Illumina).

### Gene set enrichment analysis (GSEA)

GSEA was performed on the results of the differential gene expression analysis using the ClusterProfiler package and the hallmark gene set from the molecular signatures database collection using the msigdbr package. In detail, the differentially expressed gene list was ranked according to the combined log fold change and adjusted p value and used as an input for the gene set enrichment analysis.

### Single-cell data analysis

We used a deconvolution method to investigate the relationship between WDR82-high tumors and neutrophils in BRCA^29^. A single-cell RNA-seq dataset of breast cancer (BRCA, https://zenodo.org/records/10672250)^30^ was used as the reference profile, in which neutrophils and tumor cells had been annotated. Based on this, we classified tumor cells into WDR82-positive (pos) and WDR82-negative (neg) subgroups according to the mean of normalized WDR82 expression, while other cell types remained unchanged. We then used InstaPrism^31^ to learn the gene expression signatures of each cell type.

### Statistical analysis

For RNA-seq data, statistical analysis was performed with R (version 3.4.0). Prism V.10.0 software (GraphPad) was used for statistical analysis. Analysis for significance was performed by two-way analysis of variance (ANOVA) when more than two groups were compared and by parametric or non-parametric Student's t-test when only two groups were compared. P value<0.05 was considered statistically significant (*p<0.05, **p<0.01, ***p<0.001, ****p<0.0001). Survival was evaluated using the Kaplan-Meier method and analyzed by the Mantel-Cox log-rank test. Unless indicated, all results were performed at least three independent experiments, and n refers to biological replicates. All data are presented as the mean±SD.

### Data and materials availability

The RNA sequencing data is deposited in the Gene Expression Omnibus under accession code GSE325774. All other study data are included in the article and supplemental information. Any additional information required to reanalyze the data reported in this paper is available from the leader contact upon request.

## Results

### Reduced expression or deficiency of *Wdr82* promotes tumor growth *in vivo*

**Figure 1:**
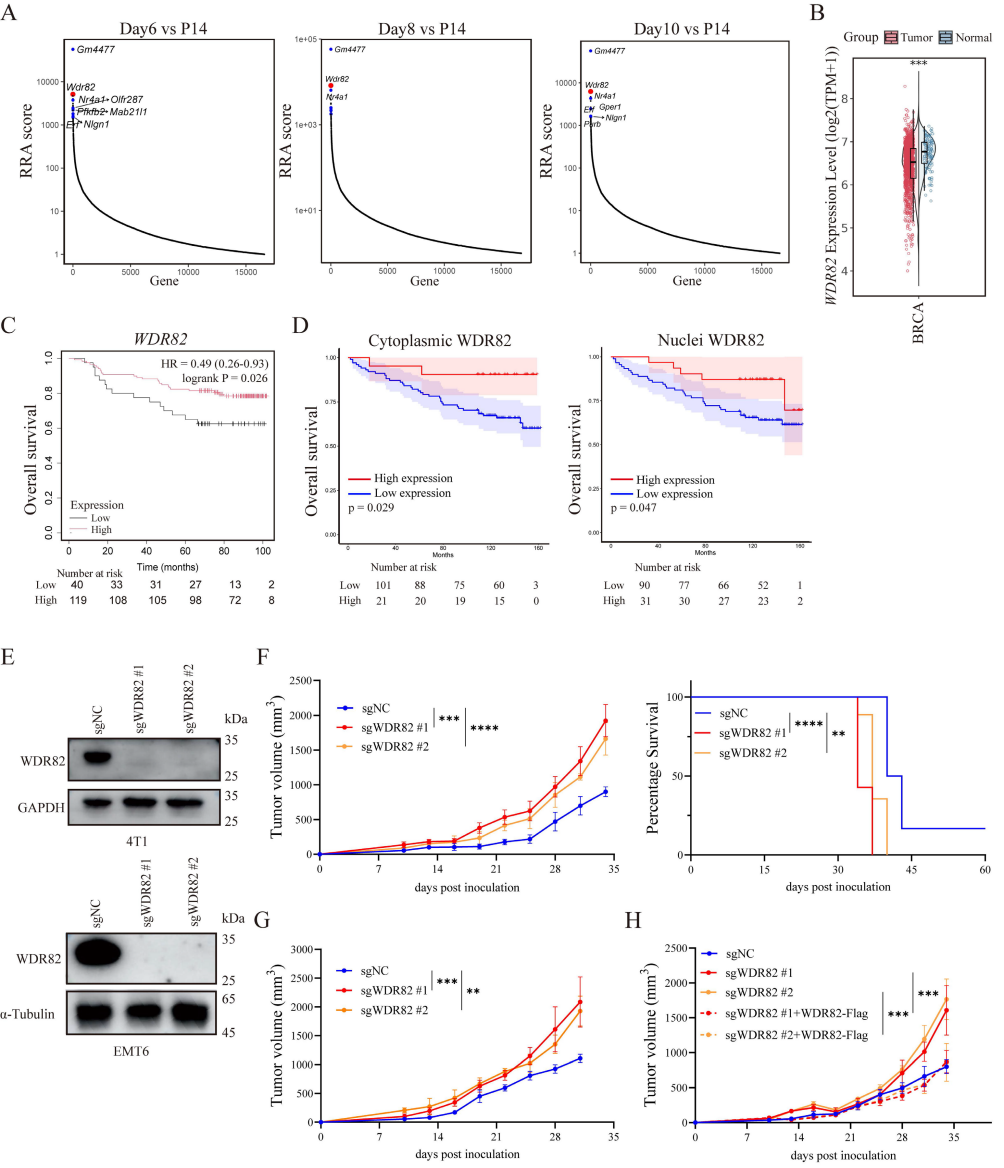
** Lacking *Wdr82* promotes tumor growth and shortens survival of tumor-bearing host.** (A) RRA score of top enriched genes in the genome-wide CRISPR-Cas9 screenings at different days after 4T1 tumor inoculation compared with 4T1 cells transduced with mGeCKOv2 after 14-day of puromycin selection *in vitro* (P14). (B) WDR82 expression difference between normal tissue and tumor tissue in the TCGA cohort. (C) Kaplan-Meier survival analysis of patients with BRCA stratified by WDR82 expression levels (75% cut-off). Overall survival was analyzed in the GSE1456 datasets. (D) Overall survival analysis of BRCA patients with low or high cytoplasmic (left) or nuclei (right) WDR82 expression levels. (E) Western blot analysis of WDR82 expression in 4T1 (up) or EMT6 (down) cells transfected with sgRNAs targeting WDR82 (sgWDR82) or empty vector (sgNC). The knockout cell lines were constructed through CRISPR-Cas9 system and the 4T1 or EMT6 cells transfected with empty vector were used as control. (F) Tumor growth (left) and survival (right) of orthotopic *Wdr82^-/-^* or control 4T1 tumors in BALB/c mice (n=5). (G) Tumor growth of orthotopic *Wdr82^-/-^* or control EMT6 tumors in BALB/c mice (n=5). (H) Tumor growth curves of orthotopic *Wdr82^-/-^, Wdr82^-/-^* transfected with pcDNA3.1-WDR82-flag (sgWDR82+WDR82-Flag) or control 4T1 tumors in BALB/c mice (n=5). Data show the mean±SD of biological replicates or are representative graphs of one (**A**) or three (**E-H**) independent experiments. Wilcox rank sum test (**B**), Two-way ANOVA (**F**-**H**) or Log-Rank test (**C, D and F**). **p<0.01, ***p<0.001, ****p<0.0001.

In our previous research, *Wdr82* was recognized as one of the top-ranked enriched genes in an *in vivo* genome-wide CRISPR-Cas9 screening of murine breast cancer model (Figure 1A)^28^, which indicated that cancer cell WDR82 suppressed tumor development. Since there were no reports on WDR82 regulating breast cancer progression, we first analyzed datasets from TCGA and found that *WDR82* expression was downregulated in breast cancer tissues, with its high expression level correlated with patients' longer survival (Figure 1B-C). We further validated this finding by analyzing an independent cohort of 129 human breast cancer tissues, which indicated low WDR82 to be associated with reduced patients' survival (Figure 1D).

To further explore the potential role of WDR82 in breast cancer, we knocked out *Wdr82* in mouse TNBC cancer cell line 4T1 and EMT6 (*Wdr82*^-/-^, Figure 1E). Using an orthotopic mouse breast cancer model, we demonstrated that tumor growth was significantly accelerated in BALB/c mice implanted with *Wdr82^-/-^* cells compared with control cells (Figure 1F-G). Moreover, *Wdr82* deficiency markedly shortened the survival time of tumor-bearing mice, which was consistent with the clinical observations in human breast cancer (Figure 1D, F). Accordingly, replenishing WDR82 expression in these knockout cells markedly reversed the tumor-promoting effect (Figure 1H). Collectively, WDR82 expression is negatively associated with tumor progression in both human clinical specimens and mouse models, indicating that WDR82 might inhibit tumor progression.

### WDR82 promotes cancer cell proliferation and invasion* in vitro*

**Figure 2:**
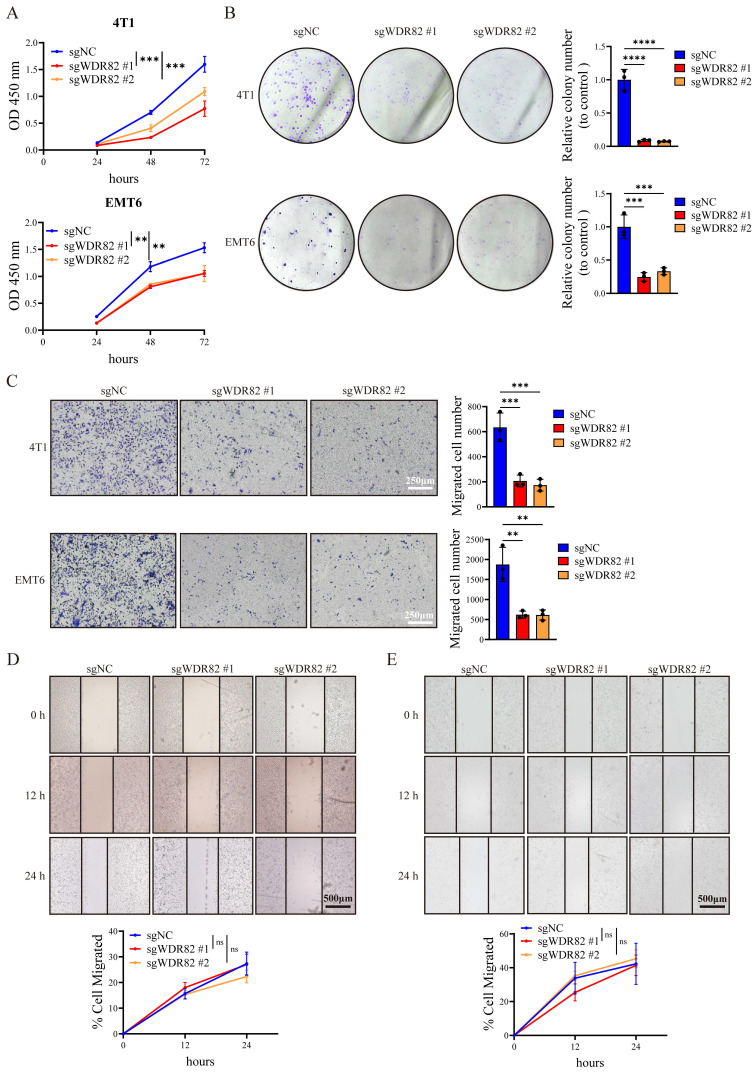
** Deficiency of WDR82 inhibits cancer cell proliferation and invasion* in vitro.*** (A) CCK8 analysis of 4T1 (up) or EMT6 (down) breast cancer cells with or without *Wdr82* deficiency (n=3). The 4T1 or EMT6 cells transfected with empty vector were used as control. (B) Colony formation assay of 4T1 (up) or EMT6 (down) breast cancer cells with or without *Wdr82* deficiency (n=3). (C) Transwell assay of 4T1 (up) or EMT6 (down) breast cancer cells with or without *Wdr82* deficiency (n=3, scale bar, 250μm). (D) Wound healing assay of *Wdr82^-/-^* or control 4T1 cells (n=3, scale bar, 500μm). (E) Wound healing assay of *Wdr82^-/-^* or control EMT6 cells (n=3, scale bar, 500μm). Data show the mean±SD of biological replicates or are representative graphs of three (**B-E**) independent experiments. Two-way ANOVA (**A, D-E**) or one-way ANOVA with Tukey's multiple comparison (**B**-**C**). ***p<0.001, ****p<0.0001.

We next explored the effect of *Wdr82* knockout on tumor cell proliferation *in vitro*. Surprisingly, CCK8 assays showed inhibited cell proliferation in *Wdr82*^-/-^ 4T1 and EMT6 cells (Figure 2A). Colony-formation assays further revealed a significant reduction in colony number in *Wdr82^-/-^* cells, indicating that *Wdr82* deletion suppressed cell growth* in vitro* (Figure 2B). We further analyzed the impact of WDR82 on cell invasion ability *in vitro*. Transwell invasion and wound-healing assays showed that *Wdr82* knockout markedly reduced the invasion of 4T1 and EMT6 cells (Figure 2C-E). Furthermore, all these effects could be attenuated through WDR82 rescue (Supplementary Figure 1). These *in vitro* assays challenged the results about the tumor-suppressive role of WDR82 observed from mouse model *in vivo*.

Taken together, our *in vitro* functional assays indicate WDR82 as a tumor-promoting molecule, whereas analyses of patient specimens and murine models yield opposing results. These findings lead us to hypothesize that the tumor-suppressive effect of WDR82 *in vivo* may rely on host immune response and the modulation of TME.

### Tumor cell deficiency of WDR82 promotes tumor progression through elevating neutrophil infiltration in TME

**Figure 3:**
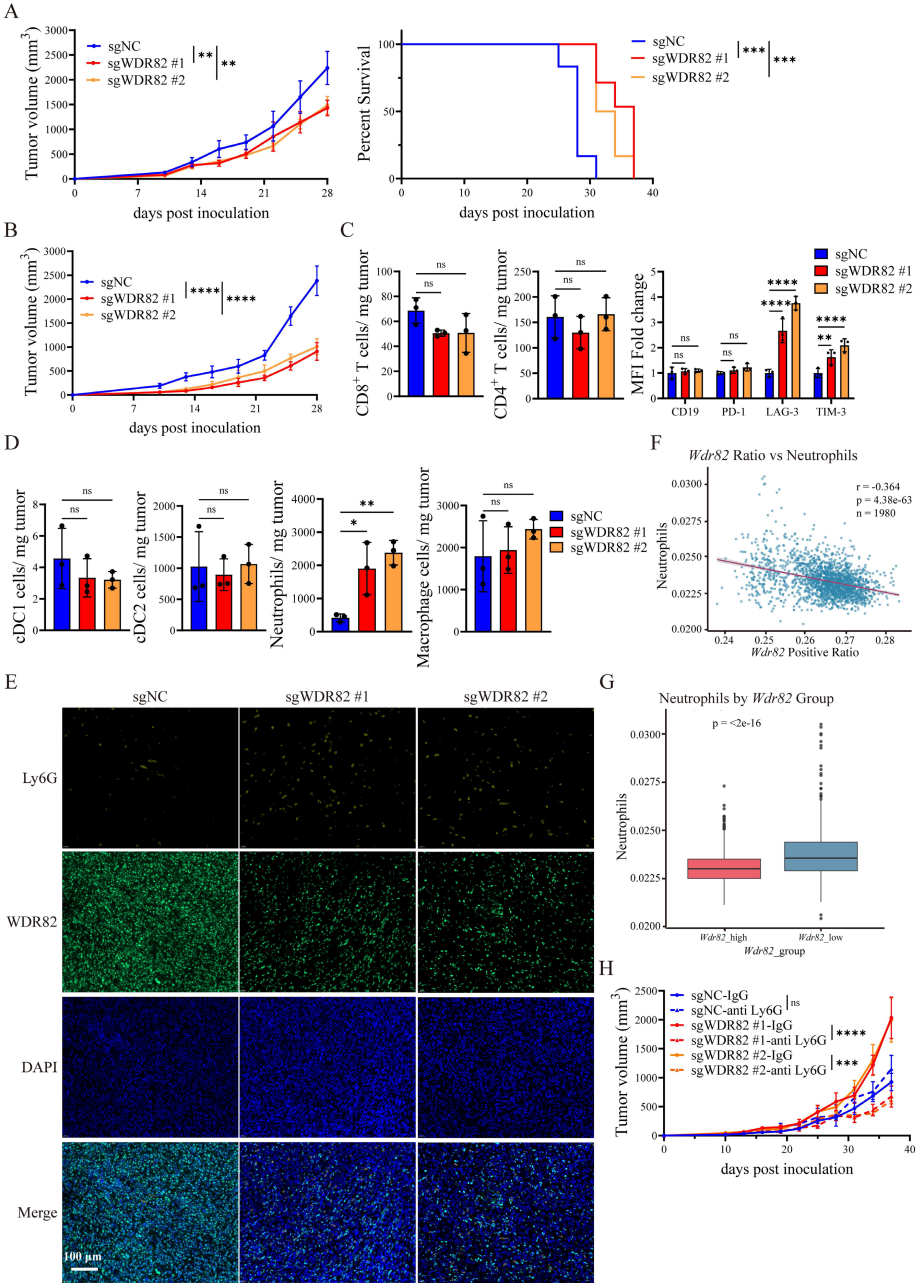
**Loss of tumor cell WDR82 promotes tumor progression via enhancing neutrophil infiltration in TME.** (A) Tumor growth (left) and survival (right) of orthotopic *Wdr82^-/-^* or control 4T1 tumors in NOD/SCID mice (n=5). The 4T1 cells transfected with empty vector were used as control. (B) Tumor growth of orthotopic *Wdr82^-/-^* or control EMT6 tumors in NOD/SCID mice (n=5). (C) Flow cytometry assay of infiltrated lymphocytes in *Wdr82^-/-^* or control 4T1 tumors in BALB/c mice. Tumors were dissected for analysis when tumor volume reached 2000mm^3^ (n=3). (D) Flow cytometry assay of infiltrated myeloid cells in *Wdr82^-/-^* or control 4T1 tumors in BALB/c mice. Tumors were dissected for analysis when tumor volume reached 2000mm^3^ (n=3). (E) mIF staining of WDR82 (green) and neutrophil (Ly6G^+^, gold) in *Wdr82^-/-^* and control 4T1 tumor samples in BALB/c mice (n=3, scale bar, 100 μm). (F) Correlation between expression level of *WDR82* and neutrophil infiltration in scRNA-seq of 119 BRCA patient samples. (G) Comparison of neutrophil infiltration proportions between the two subgroups classified by deconvolution using bulk RNA-seq data of over 1900 BRCA patient samples. (H) Tumor growth curves of orthotopic *Wdr82^-/-^* or control 4T1 tumors in BALB/c mice treated with anti-IgG antibody or anti-Ly6G antibody (n=5). Data show the mean±SD of biological replicates or are representative graphs of one (**E**) or three (**A-B, H**) independent experiments. Two-way ANOVA (**A-B, H**), one-way ANOVA with Tukey's multiple comparison (**C**-**D**) or Log-Rank test (**A**). **p<0.01, ***p<0.001, ****p<0.0001, ns, not significant.

Notably, we found that *Wdr82^-/-^* breast cancer cells exhibited accelerated tumor progression in immunocompetent BALB/c mice (Figure 1F-G), but exhibited impaired tumor growth once inoculated into NOD/SCID immunodeficient mice (Figure 3A-B), which matches the observation about the inhibited proliferation of *Wdr82^-/-^* breast cancer cells *in vitro* and further suggests the *in vivo* tumor-promoting effect of *Wdr82* deficiency in tumor cells is perhaps through the interaction of tumor-host immune system.

We next characterized the immune cell composition in *Wdr82^-/-^* and control 4T1 tumors (Supplementary Figure 2A-C). The flow cytometry analysis revealed a significant increase in neutrophil infiltration (defined as Ly6G⁺CD11b⁺ cells) and CD8^+^ T cell exhaustion, as evidenced by elevated expression of the exhaustion marker LAG-3 and TIM-3 on CD8^+^ T cells (Figure 3C-D). Since the increased exhaustion in T cells might be a result of elevated neutrophils^29^, ^32^, we focused on neutrophil infiltration and confirmed the increased accumulation of neutrophils in *Wdr82^-/-^* tumors by mIF staining (Figure 3E).

To extend these observations to human breast cancer, we analyzed single-cell RNA sequencing (scRNA-seq) data from breast cancer patients^30^. Consistent with the mouse model results, *WDR82* expression was negatively correlated with neutrophil infiltration in human breast tumor tissues (Figure 3F). In addition, analysis of a Bulk RNA-seq data (https://www.cbioportal.org/study/plots?id=brca_metabric) indicated increased neutrophil infiltration in WDR82 low-expressing tumors, supporting the clinical relevance of the WDR82-neutrophil regulatory axis (Figure 3G).

To directly verify the indispensable role of neutrophils in mediating the *in vivo* tumor-promoting effect of cancer cell *Wdr82* deficiency, we performed neutrophil depletion experiments by injecting anti-Ly6G neutralizing antibodies (or IgG isotype control) into BALB/c mice bearing *Wdr82^-/-^* or control tumors. Notably, neutrophil depletion significantly inhibited tumor growth compared with the isotype control (Figure 3H, Supplementary Figure 2D). Importantly, depletion of neutrophils in *Wdr82^-/-^* tumor-bearing mice reduced tumor growth to a level even lower than that of control tumors, which recapitulated the suppressed tumor growth phenotype of *Wdr82* deficiency observed in NOD/SCID mice. These results suggest WDR82 functions as a tumor suppressor *in vivo* mainly through suppression of neutrophils in the interaction of tumor-host immune system. *In vivo* depletion of neutrophils impaired tumor-promoting effect of *Wdr82* deficiency, even made it show tumor-suppressing effect as compared to control, which to some content aligned with the inhibited cell proliferation and migration of *Wdr82* deficient tumor cells *in vitro*.

Taken together, these data demonstrate that tumor cell deficiency of *Wdr82* promotes tumor progression by enhancing neutrophil infiltration into the TME, which in turn creates an immunosuppressive TME that favors tumor growth.

### WDR82 deficiency promotes neutrophil infiltration through elevating tumor cell expression of CXCL7 and CXCL2

**Figure 4:**
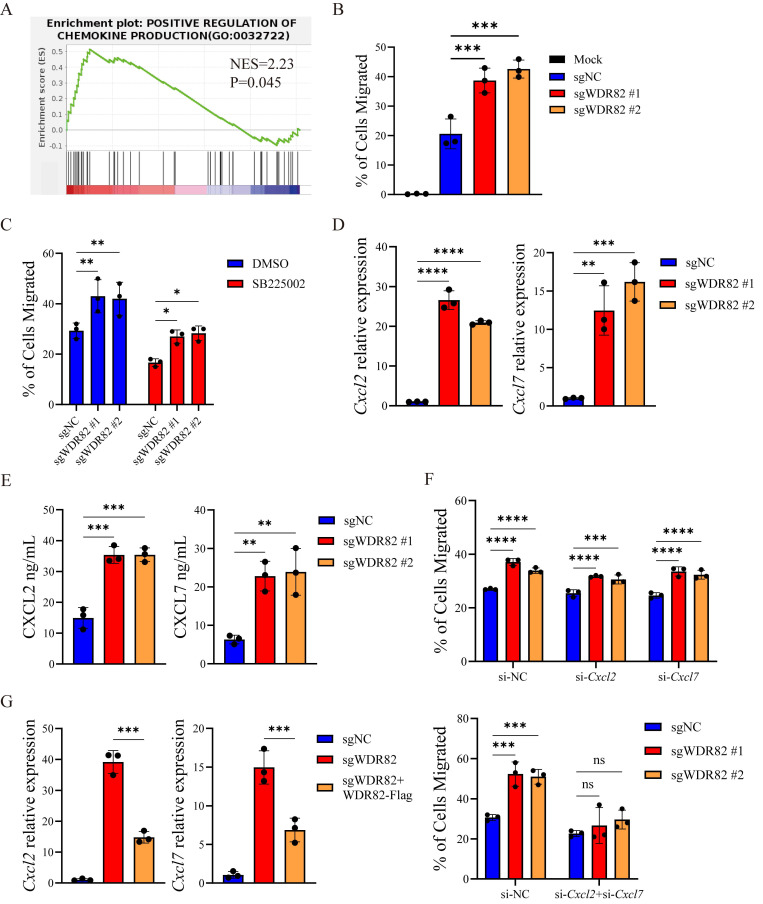
** WDR82 deficiency enhances neutrophil infiltration through elevating tumor cell expression of CXCL2 and CXCL7.** (A) GSEA showing enrichment of gene signatures in bulk RNA-seq analysis of 4T1 cells with or without *Wdr82* deficiency (n=3). (B) Percentage of cells migrated to the lower chamber when CM from *Wdr82^-/-^* or control 4T1 cells was added (n=3). The 4T1 cells transfected with empty vector were used as control. Mock, RPMI-1640 medium supplemented with 10% FBS and 1% penicillin-streptomycin. (C) Percentage of DMSO or SB225002 (100nM, 1 hour) pre-treated cells migrated to the lower chamber when CM from *Wdr82^-/-^* or control 4T1 cells supplemented with DMSO or 100nM SB225002 was added (n=3). (D) qRT-PCR analysis of *Cxcl2* (left) or *Cxcl7* (right) expression in *Wdr82^-/-^* or control 4T1 cells (n=3). (E) ELISA of CXCL2 (left) or CXCL7 (right) secretion in *Wdr82^-/-^* or control 4T1 cells (n=3). (F) Percentage of cells migrated to the lower chamber when CM from *Wdr82^-/-^* or control 4T1 cells transfected with si-NC, si-*Cxcl2*/si-*Cxcl7* alone (up) or concurrently (down) was added (n=3). (G) qRT-PCR analysis of *Cxcl2* (left) or *Cxcl7* (right) expression in *Wdr82^-/-^* or control 4T1 cells transfected with pcDNA3.1-WDR82-flag or pcDNA3.1 empty vector (n=3). Data show the mean±SD of biological replicates. One-way ANOVA with Tukey's multiple comparison. *p<0.05, **p<0.01, ***p<0.001, ****p<0.0001, ns, not significant.

To elucidate the molecular mechanism by which *Wdr82* deficiency enhances TME neutrophil infiltration, we performed bulk RNA-seq on *Wdr82^-/-^* and control 4T1 cells (Supplementary Figure 3A-B). GSEA enrichment revealed that the chemokine production pathway was significantly upregulated in *Wdr82^-/-^* cells (Figure 4A), suggesting that *Wdr82* knockout might promote neutrophil recruitment by enhancing chemokine secretion.

To validate this hypothesis, we performed neutrophil migration transwell assays using conditioned media (CM) from *Wdr82^-/-^* or control 4T1 cells. Consistent with the RNA-seq results, CM from *Wdr82^-/-^* cells significantly increased neutrophil migration compared with that from control cells (Figure 4B). Notably, pre-treating neutrophils with SB225002 (a specific CXCR2 inhibitor) abrogated this enhanced migration (Figure 4C), indicating that the chemotactic effect of *Wdr82^-/-^* cells-derived CM was dependent on CXCR2 signaling—an axis critical for neutrophil recruitment^33^, ^34^.

We next sought to identify the key chemokines upregulated in *Wdr82^-/-^* cells. qRT-PCR analysis confirmed that the mRNA levels of *Cxcl2* and *Cxcl7* were significantly increased in *Wdr82^-/-^* cells (Figure 4D, Supplementary Figure 3C). These findings were further validated by ELISA assay, which demonstrated elevated secretion of CXCL2 and CXCL7 in *Wdr82^-/-^* cells (Figure 4E).

To directly confirm the functional role of CXCL2 and CXCL7 in *Wdr82^-/-^*-mediated neutrophil recruitment, we knocked down *Cxcl2* and *Cxcl7* respectively or concurrently in *Wdr82^-/-^* cells using targeted siRNAs (si-*Cxcl2,* si-*Cxcl7 or* si-*Cxcl2* + si-*Cxcl7*). Transwell assays showed that siRNA-mediated silencing of *Cxcl2* or *Cxcl7* alone had mild effect on neutrophil migration, while simultaneously silencing of *Cxcl2* and *Cxcl7* significantly reduced neutrophil migration induced by *Wdr82^-/-^* cell CM (Figure 4F, Supplementary Figure 3D-E). This confirmed that CXCL2 and CXCL7 were the key chemokines responsible for enhanced neutrophil recruitment in the context of *Wdr82* deficiency. Finally, we performed a rescue experiment to verify that the upregulation of CXCL2 and CXCL7 was directly caused by *Wdr82* depletion. Re-expression of WDR82 in *Wdr82^-/-^* cells markedly reduced the mRNA levels of *Cxcl2* and *Cxcl7* (Figure 4G).

Collectively, these data demonstrate that *Wdr82* knockout directly upregulates the expression and secretion of CXCL2 and CXCL7 by tumor cells, which in turn promotes neutrophil migration via CXCR2 signaling, ultimately contributing to tumor progression.

### WDR82 deficiency promotes tumor cell CXCL7 and CXCL2 expression by elevating ERK activation

**Figure 5:**
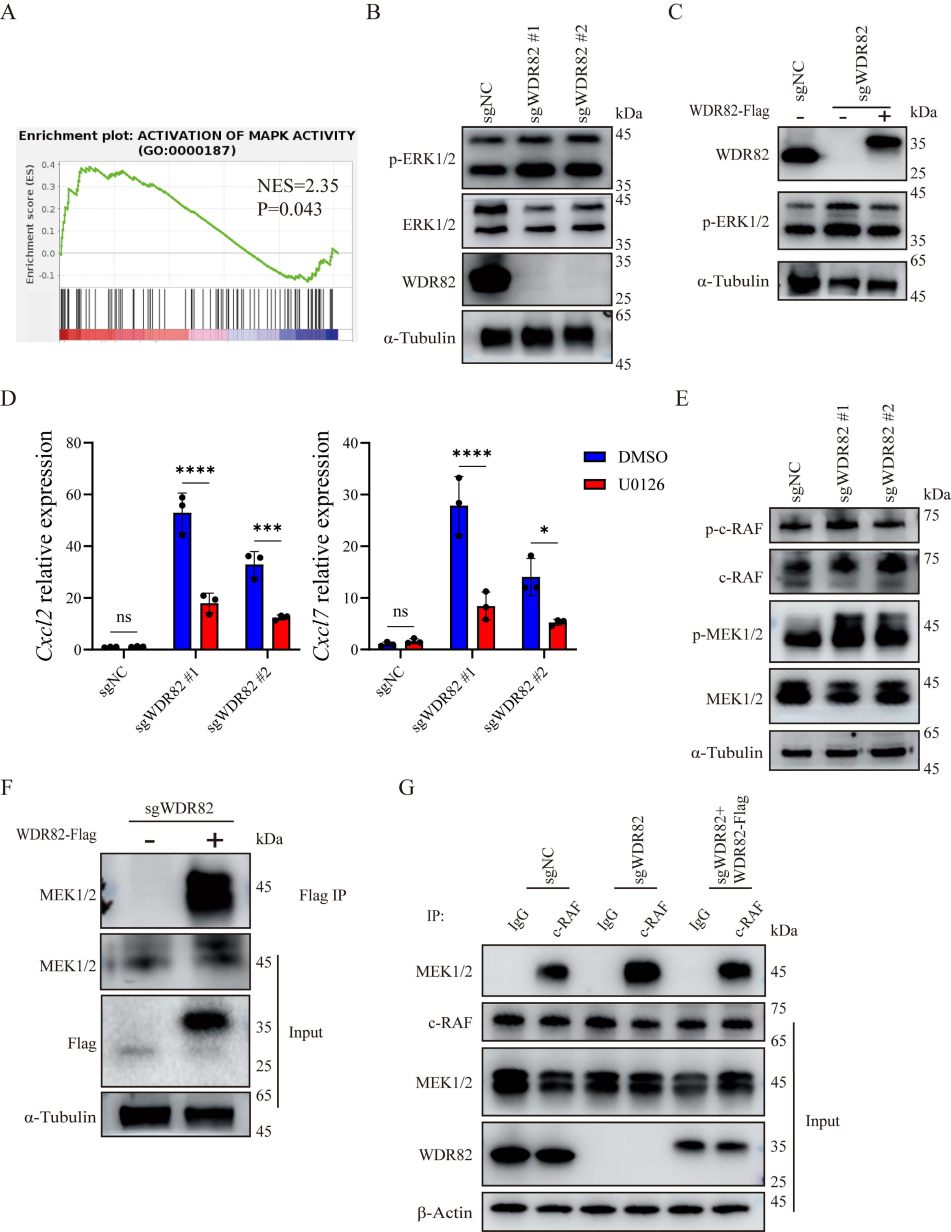
** Loss of WDR82 promotes CXCL7 and CXCL2 expression by activating ERK.** (A) GSEA showing enrichment of gene signatures in bulk RNA-seq analysis of 4T1 cell with or without *Wdr82* deficiency (n=3). (B) Western blot analysis of indicated proteins in *Wdr82^-/-^* or control 4T1 cells. The 4T1 cells transfected with empty vector were used as control. (C) Western blot analysis of indicated proteins in *Wdr82^-/-,^ Wdr82^-/-^*+WDR82-Flag or control 4T1 cells. (D) qRT-PCR analysis of *Cxcl2* (left) or *Cxcl7* (right) expression in *Wdr82^-/-^* or control 4T1 cells treated with DMSO or 20 μM U0126 for 1 hour (n=3). (E) Western blot analysis of indicated proteins in *Wdr82^-/-^* or control 4T1 cells. (F) Co-immunoprecipitation of WDR82 with MEK1/2. (G) Coimmunoprecipitation of c-RAF with MEK1/2. Data show the mean±SD of biological replicates or are representative graphs of three (**B-C, E-G**) independent experiments. Two-way ANOVA (**D**). ***p<0.001, ****p<0.0001.

GSEA of bulk RNA-seq data indicated that ERK activation was significantly upregulated in *Wdr82* ablated cells (Figure 5A). Both CXCL2 and CXCL7 expression can be regulated via the ERK signaling pathway^35^. Accordingly, we observed elevated ERK phosphorylation in *Wdr82^-/-^* cells, an effect that was notably attenuated upon WDR82 reconstitution (Figure 5B-C). Thus, *Wdr82* deficiency led to enhanced ERK signaling in tumor cells.

To establish the functional link between the increased ERK phosphorylation and the upregulation of CXCL2 and CXCL7, we treated cells with U0126, a specific inhibitor of ERK phosphorylation. We found that inhibition of ERK signaling markedly blunted the induction of *Cxcl2* and *Cxcl7* in *Wdr82^-/-^* cells (Figure 5D, Supplementary Figure 3F). These results confirmed that ERK activation contributes to *Wdr82* ablation induced CXCL2 and CXCL7 expression. Further evaluation of upstream ERK pathway components revealed increased phosphorylation of MEK1/2, whereas the phosphorylation level of c-RAF remained unchanged (Figure 5E). These results suggest that WDR82 inhibited ERK phosphorylation through suppressing MEK1/2 activation.

WDR83, a structural homolog of WDR82, has been reported to directly bind to MEK1/2 and promote ERK signaling^36^. We wondered whether WDR82 might function in the same way. Subsequently, co-IP assays validated a direct interaction between WDR82 and MEK1/2 (Figure 5F). Furthermore, co-IP also indicated an increased association between MEK1/2 and c-RAF in *Wdr82^-/-^* cells, which could be inhibited by WDR82 replenishing (Figure 5G). Collectively, these results demonstrate that WDR82 binds to the ERK upstream kinase MEK1/2 and consequently disturbs the assembly of ERK signaling proteins, resulting in restrained ERK activation. Loss of WDR82 relieves this inhibitory constraint, leading to hyperactivated ERK signaling which in turn drives the increased production of CXCL2 and CXCL7 and subsequent neutrophil infiltration.

### Adenovirus-mediated intratumoral overexpression of WDR82 suppresses tumor growth by remodeling TME

**Figure 6:**
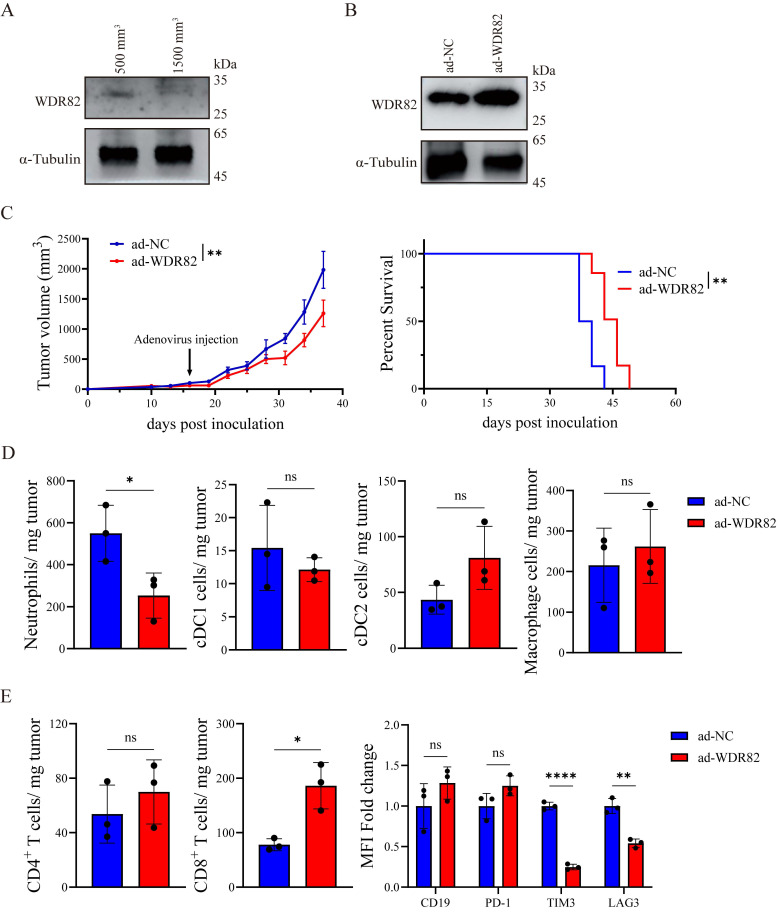
** Intratumoral overexpression of WDR82 by adenovirus delivery remodels the TME and suppresses tumor growth.** (A) Western blot analysis of WDR82 levels in CD45^-^ CD326^+^ cells isolated from orthotopic 4T1 tumors at indicated tumor volume. (B) Western blot analysis of WDR82 levels in 4T1 cells transfected with negative control adenovirus (ad-NC) or WDR82-expressing adenovirus (ad-WDR82). (C) Tumor growth (left) and survival (right) of 4T1-bearing mice with intratumoral injection of ad-WDR82 or ad-NC when tumor volume reached 100mm^3^. (D) Flow cytometry analysis of myeloid cells infiltration in tumors from 4T1-bearing mice with intratumoral injection of ad-WDR82 or ad-NC. Tumors were dissected when tumor volume reached 2000mm^3^ (n=3). (E) Flow cytometry analysis of lymphocytes infiltration in tumors from 4T1-bearing mice with intratumoral injection of ad-WDR82 or ad-NC. Tumors were dissected when tumor volume reached 2000mm^3^ (n=3). Data show the mean±SD of biological replicates or are representative graphs of three independent experiments. Unpaired two-tailed Student's t-test (**C**-**E**) or Log-Rank test (**C**). *p<0.05, **p<0.01, ns, not significant.

As previously demonstrated, WDR82 expression was downregulated in tumor tissues as tumor advanced (Figure 6A), which might represent a potential therapeutic target for cancer treatment. We thus investigated whether intratumoral overexpression of WDR82 could suppress tumor growth *in vivo*. We generated a WDR82-expressing adenovirus (ad-WDR82) and found that adenovirus-mediated intratumoral WDR82 overexpression markedly inhibited tumor growth and prolonged survival of breast tumor-bearing mice (Figure 6B-C). Notably, flow cytometry analysis revealed reduced neutrophil infiltration in tumors treated with Ad-WDR82 (Figure 6D), supporting our hypothesis that WDR82 modulated tumor growth by regulating neutrophil infiltration. It was reported that neutrophils promoted the exhaustion of CD8^+^ T cells, thereby facilitating the formation of an immune suppressive TME. We therefore investigated the effect of adenovirus-mediated intratumoral overexpression of WDR82 on immune cell composition of the TME, and found that CD8^+^ T cell infiltration was elevated and exhaustion was decreased after Ad-WDR82 intratumoral treatment (Figure 6E). These results suggest an immune-promoting role of intratumoral WDR82 overexpression in remodeling the immunosuppressive TME.

Therefore, adenovirus-mediated intratumoral overexpression of WDR82 suppresses tumor growth by inhibiting neutrophil infiltration, highlighting the potential of WDR82 as a promising target for cancer immunotherapy.

## Discussion

In this study, we uncover a previously unrecognized tumor-suppressive mechanism by which WDR82 safeguards against breast cancer progression through restraining tumor cell ERK-mediated chemokine production and subsequent neutrophil infiltration in TME. Our findings challenge the conventional view that tumor suppressors primarily act cell-autonomously to inhibit proliferation: despite suppressing breast cancer cell growth *in vitro*, *Wdr82* deficiency unexpectedly accelerated tumor growth *in vivo* by promoting neutrophil infiltration and remodeling the TME towards an immunosuppressive state. This paradoxical observation underscores a critical conceptual shift: tumor suppression must be understood not only at the level of cancer cell-intrinsic control, but also through the lens of host immune modulation. WDR82 exemplifies this paradigm, functioning as a gatekeeper that prevents the establishment of a pro-tumorigenic niche by limiting neutrophil recruitment in TME.

Tumor-infiltrating immune cells play a crucial role in tumor progression. Neutrophils are usually the most abundant immune cell type in the TME and are usually correlated with poor survival and therapeutic outcomes^37^. Bulk RNA-seq verified increased chemokine production in *Wdr82^-/-^* 4T1 cells, leading us to focus on the chemotaxis of neutrophils. Our findings demonstrated that *Wdr82* deficiency significantly increased neutrophil infiltration in TME, providing new insights into the regulation of tumor-associated neutrophil recruitment.

While CXCL2 and CXCL7 are well-established neutrophil chemoattractants acting through CXCR2^38^-^40^, the upstream mechanisms controlling their expression in cancer cells remain incompletely defined. Here we demonstrate that ERK activation downstream of WDR82 loss drives CXCL2/CXCL7 production, establishing a direct link between a tumor suppressor and chemokine-mediated immune infiltration. Mechanistically, CXCLs expression can be activated through various signaling pathways, such as ERK signaling pathway, caspase-3 pathway and PI3K signaling pathway^5^, ^41^, ^42^. By analyzing RNA-seq data, we identified increased ERK activation. Further investigation showed that WDR82 depletion significantly increased ERK and MEK1/2 phosphorylation but had no effect on c-RAF activation. This effect was abolished when WDR82 was replenished into the knockout cells. Inhibiting ERK activation with U0126 significantly inhibited CXCL2 and CXCL7 expression, proving that ERK pathway activation participates in *Wdr82* knockout-induced chemokine production. Notably, other ERK-regulated chemokines such as CXCL1 and CXCL5 were not upregulated in *Wdr82*-deficient cells, suggesting context-specific transcriptional control that may involve additional epigenetic or transcriptional co-factors. This selectivity raises the intriguing possibility that WDR82 governs a specific chemokine program rather than broadly modulating ERK-dependent gene expression, a hypothesis that merits future investigation into the chromatin landscape and transcription factor occupancy at these chemokine loci.

Apart from tumor cells, macrophages are another important source of CXCL2 in the TME. On one hand, tumor cells decrease WDR82 expression to increase CXCL2 secretion, thereby potentially recruiting macrophages and facilitating its differentiation into M2 macrophages to suppress immunity^43^. On the other hand, the infiltrated neutrophils may also improve macrophage migration and differentiation while the infiltrated macrophages in turn secret CXCL2 to recruit neutrophils, forming a positive feedback loop^23^, ^44^, ^45^. When WDR82 was overexpressed in tumor cells, it breaks the loop and inhibits neutrophil infiltration to form an immunosuppressive TME and consequently inhibit tumor growth.

WDR82 is first identified as a component of the SET1/COMPASS complex that regulates histone methylation and gene transcription^12^, ^46^. Its roles in cancer progression has been rarely investigated, although most current studies suggest that it is aberrantly regulated in tumor tissues. In our study, we identify WDR82 as a novel negative regulator of the ERK signaling cascade. Considering that WDR82 depletion had a mild effect on c-RAF phosphorylation, we focused on the interaction between MEK1/2 and WDR82. With its structural homolog WDR83 (MORG1) scaffolds MEK and ERK to facilitate signal transduction^36^, ^47^, ^48^, we tested whether WDR82 may function in the same way. We discovered WDR82 binds directly to MEK1/2 and disrupts its interaction with c-RAF, subsequently imposing a brake on ERK activation. Therefore, depleting WDR82 eliminated its inhibition and in turn promoted ERK activation, leading to increased chemokine production and neutrophil infiltration. This functional antagonism between two structurally related WD-40 proteins reveals an elegant regulatory logic: the same signaling pathway can be fine-tuned by opposing scaffold-like molecules that either promote or restrain kinase assembly. This finding adds a new layer of complexity to our understanding of ERK pathway regulation^49^ and suggests that the balance between WDR82 and WDR83 may determine the amplitude and duration of ERK signaling in cancer cells. Whether this balance is perturbed in breast cancer and whether it can be therapeutically exploited warrants further investigation.

From a therapeutic perspective, our study positions WDR82 as a promising target for cancer immunotherapy. The finding that intratumoral adenovirus-mediated WDR82 overexpression suppresses tumor growth and reduces neutrophil infiltration demonstrates the feasibility of restoring WDR82 function as a therapeutic strategy. Importantly, WDR82 overexpression not only dampens neutrophil recruitment but also alleviates CD8⁺ T cell exhaustion, suggesting that WDR82-based therapies could synergize with immune checkpoint blockade or adoptive cell therapies by remodeling the immunosuppressive TME^50^, ^51^. Given the emerging role of neutrophils in resistance to anti-PD-1/PD-L1 therapy and CAR-T cell therapy^29^, ^52^, ^53^, combining WDR82 restoration with these immunotherapies may represent a powerful approach to overcome treatment resistance.

Several important questions remain. Firstly, what determines the selectivity of CXCL2/CXCL7 upregulation upon ERK hyperactivation? CXCL2 and CXCL7 are not the only chemokines regulated by ERK signaling, so why was there no increase in other CXCLs? Secondly, what is the structural basis for the opposing functions of WDR82 and WDR83, and can small molecules or peptides be designed to disrupt WDR83-mediated scaffolding or enhance WDR82-mediated inhibition? In addition, inhibiting ERK activation did not inhibit CXCL2/CXCL7 expression to the same level as that in control cells, indicating that there may be other mechanisms involved in the regulatory process. Thirdly, does WDR82 regulate other aspects of neutrophil biology beyond chemotaxis, such as polarization toward the N2 phenotype or the formation of neutrophil extracellular traps (NETs)? Addressing these questions will not only deepen our understanding of tumor-immune crosstalk but also pave the way for novel therapeutic interventions.

In summary, our study identifies WDR82 as a critical tumor suppressor that orchestrates immune surveillance by restraining the ERK-chemokine-neutrophil axis in TME. By uncovering this previously unknown mechanism, we provide a conceptual framework for targeting neutrophil-mediated immunosuppression and offer a rationale for developing WDR82-based therapies for breast cancer treatment.

## Supplementary Material

Supplementary figures and tables.
