# Supplementary material for: WDR82 suppresses breast cancer progression by inhibiting ERK-driven chemokine expression and neutrophil infiltration
Source: Int J Biol Sci. 2026 May 29;22(11):5878–94. doi: 10.7150/ijbs.135015 (PMC13282738; doi:10.7150/ijbs.135015)
Supplement: Supplementary file 1 — Supplementary figures and tables. [file ijbsv22p5878s1.pdf]

WDR82 suppresses breast cancer progression by inhibiting ERK-driven chemokine expression and neutrophil infiltration

Qinyi Yu, Jiang Yang, Fengjiao Lu, Wenxiu Liu, Zeyao Han, Yuanchao Bao, Xiaorui Xu, Jialei Xu, Wanfeng Gao, Boyi Cong, Yangyang Chai, Xuetao Cao*

Supplementary Figure 1-3

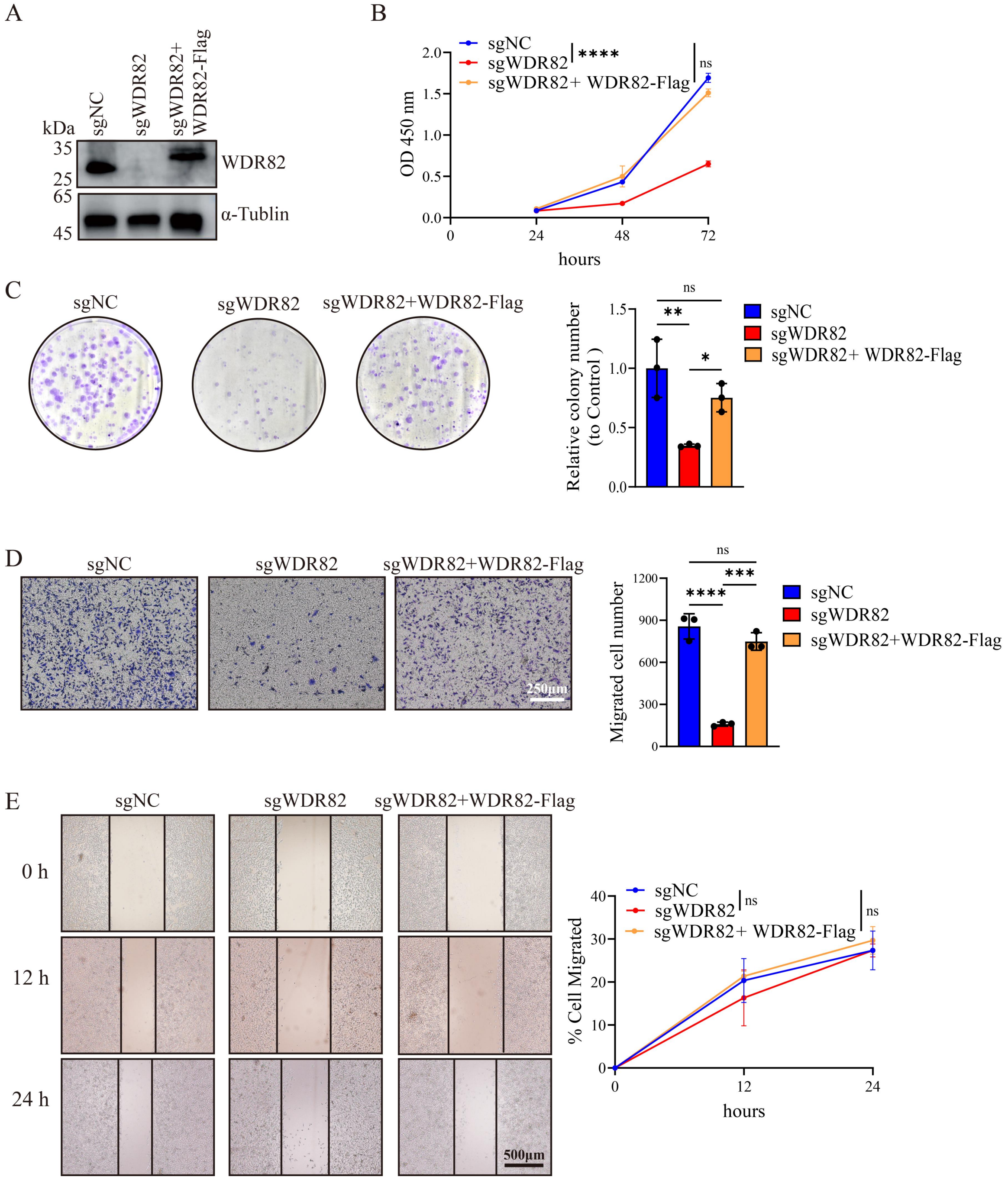

Supplementary Figure 1

***Wdr82* ablation in 4T1 cell inhibits cell proliferation and invasion *in vitro*.** (A) WB analysis of WDR82 expression in *Wdr82*$^{-/-}$ (sgWDR82), *Wdr82*$^{-/-}$ transfected with pcDNA3.1-WDR82-flag (sgWDR82+WDR82-Flag) or control (sgNC) 4T1 cells. The 4T1 cells transfected with empty vector were used as control. (B) CCK8 analysis of indicated cells (n=3). (C) Colony formation of indicated cells (n=3). (D) Transwell

assay of indicated cells (n=3, scale bar, 250μm). (E) Wound healing assay of indicated cells (n=3, scale bar, 500μm). Data show the mean ± SD of biological replicates or are representative graphs of three (**A**, **C-E**) independent experiments. Two-way ANOVA (**B, E**) or one-way ANOVA with Tukey's multiple comparison (**C**-**D**). **p<0.01, ***p<0.001, ****p<0.0001, ns, not significant.

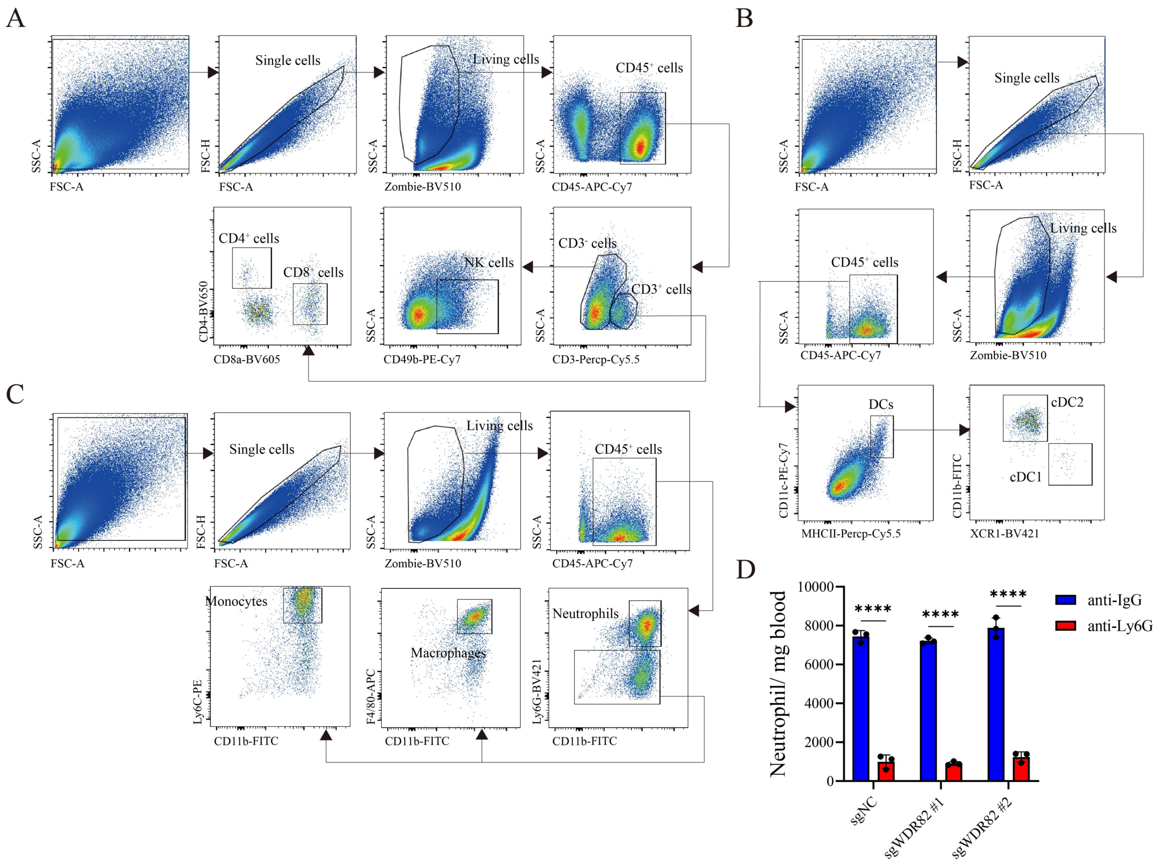

Supplementary Figure 2

***Wdr82* loss promotes tumor progression through elevating neutrophil infiltration.**

(A-C) The gating strategy for analyzing tumor infiltrating lymphoid cells (A), DCs (B) and myeloid cells (C). (D) Number of neutrophils in peripheral blood from mice with indicated treatment (n=3). Data show the mean ± SD of biological replicates. Two-way ANOVA (**D**). ****p<0.0001.

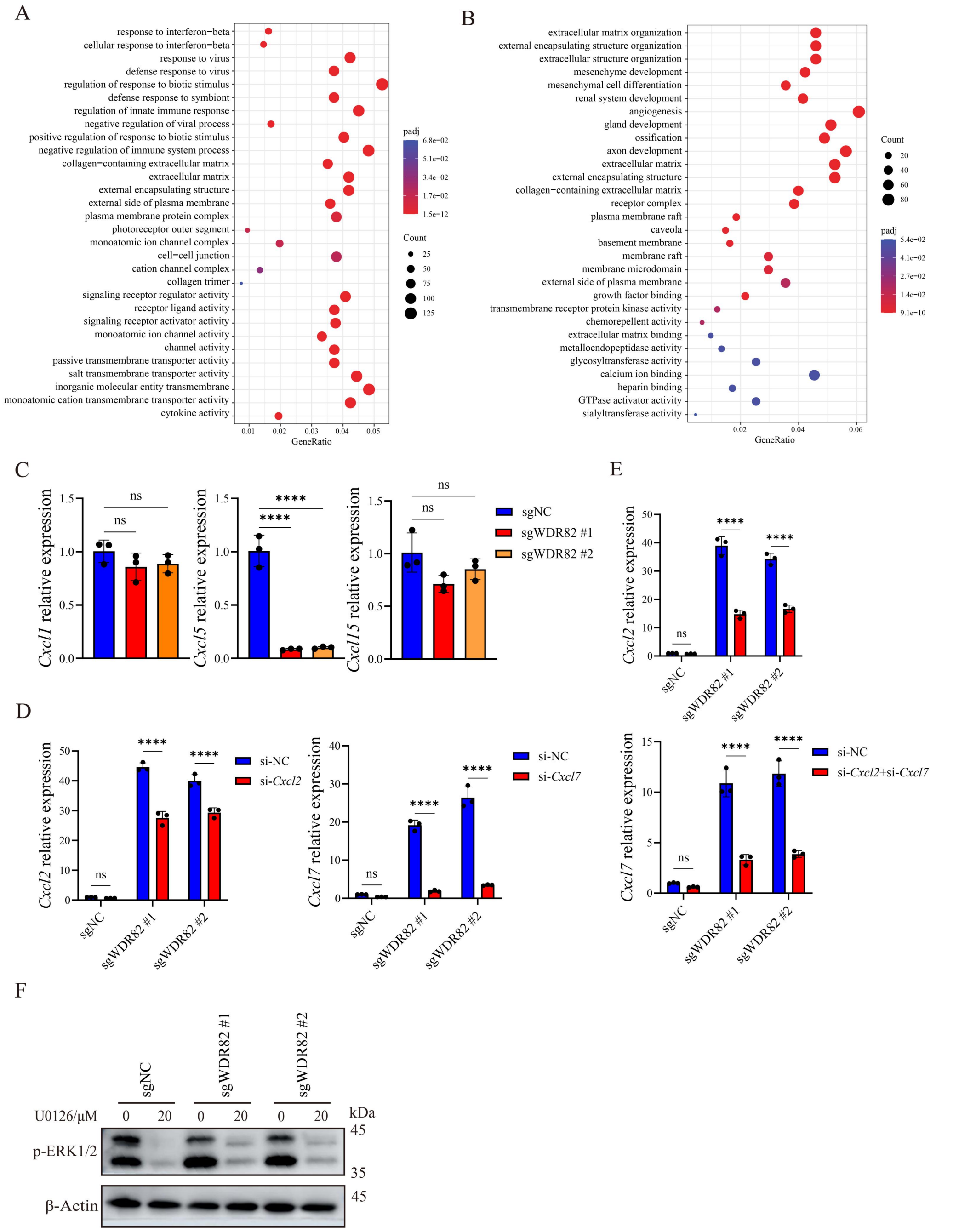

Supplementary Figure 3

Wdr82 **knockout promotes CXCL2/CXCL7 expression through increasing ERK activation.** (A) GO analysis showing enrichment of up-regulated genes in *Wdr82*$^{-/-}$ 4T1 cells from bulk RNA-seq analysis (n=3). (B) GO analysis showing enrichment of down-regulated genes in *Wdr82*$^{-/-}$ 4T1 cells from bulk RNA-seq analysis (n=3). (C) qRT-PCR analysis of *Cxcl1* (left), *Cxcl5* (middle) or *Cxcl15* (right) expression in *Wdr82*$^{-/-}$ or Control 4T1 cells (n=3). (D) qRT-PCR analysis of *Cxcl2* (left) or *Cxcl7*

(right) expression in *Wdr82*$^{-/-}$ or control 4T1 cells transfected with si-NC, si-*Cxcl2* or si-*Cxcl7* (n=3). (E) qRT-PCR analysis of *Cxcl2* (up) or *Cxcl7* (down) expression in *Wdr82*$^{-/-}$ or control 4T1 cells transfected with si-NC or si-*Cxcl2* + si-*Cxcl7* (n=3). (F) WB analysis of ERK phosphorylation in *Wdr82*$^{-/-}$ or control 4T1 cells pre-treated with U0126 or DMSO for 1 hour followed by EGF treatment. Data show the mean ± SD of biological replicates or are representative graphs of three (**F**) independent experiments. One-way ANOVA with Tukey's multiple comparison (**C**) or two-way ANOVA (**D-E**). ****p<0.0001, ns, not significant.

Supplementary Table 1-2

Supplementary Table 1 sgRNAs used for *Wdr82* knockout

Gene name	Oligonucleotides
Wdr82-sgRNA-1	Fw: 5'-CACCAACTGCCAGGTAATGGTACC-3'<br>Rv: 5'-AAACGGTACCATTACCTGGCAGTT-3'
Wdr82-sgRNA-2	Fw: 5'-CACCTAGGGCCTCATGCATCTACA-3'<br>Rv: 5'-AAACTGTAGATGCATGAGGCCCTA-3'

Supplementary Table 2 Primers used for qRT-PCR assays

Gene name	Oligonucleotides
Actb	Fw: 5'-GGCTGTATTCCCCTCCATCG-3'<br>Rv: 5'-CCAGTTGGTAACAATGCCATGT-3'
Cxcl1	Fw: 5'-CTGGGATTCACCTCAAGAACATC-3'<br>Rv: 5'-CAGGGTCAAGGCAAGCCTC-3'
Cxcl2	Fw: 5'-CCAACCACCAGGCTACAGG-3'<br>Rv: 5'-GCGTCACACTCAAGCTCTG-3'
Cxcl5	Fw: 5'-TCCAGCTCGCCATTCATGC-3'<br>Rv: 5'-TTGCGGCTATGACTGAGGAAG-3'
Cxcl7	Fw: 5'-CTCAGACCTACATCGTCCTGC-3'<br>Rv: 5'-GTGGCTATCACTTCCACATCAG-3'
Cxcl15	Fw: 5'-CAAGGCTGGTCCATGCTCC-3'<br>Rv: 5'-TGCTATCACTTCCTTTCTGTTGC-3'
Wdr82	Fw: 5'-GTGTTCCGCGAGAACTCAGAC-3'<br>Rv: 5'-TCATAGAGCACGATGGAGTCA-3'
